# LRRK2 Kinase Activity Is Dependent on LRRK2 GTP Binding Capacity but Independent of LRRK2 GTP Binding

**DOI:** 10.1371/journal.pone.0023207

**Published:** 2011-08-12

**Authors:** Jean-Marc Taymans, Renée Vancraenenbroeck, Petri Ollikainen, Alexandra Beilina, Evy Lobbestael, Marc De Maeyer, Veerle Baekelandt, Mark R. Cookson

**Affiliations:** 1 Laboratory for Neurobiology and Gene Therapy, Katholieke Universiteit Leuven, Leuven, Belgium; 2 Laboratory for biomolecular modeling, Katholieke Universiteit Leuven, Heverlee, Belgium; 3 Cell Biology and Gene Expression Section, Laboratory of Neurogenetics, National Institute on Aging (NIA), National Institutes of Health, Bethesda, Maryland, United States of America; Hertie Institute for Clinical Brain Research and German Center for Neurodegenerative Diseases, Germany

## Abstract

Leucine rich repeat kinase 2 (*LRRK2*) is a Parkinson's disease (PD) gene that encodes a large multidomain protein including both a GTPase and a kinase domain. GTPases often regulate kinases within signal transduction cascades, where GTPases act as molecular switches cycling between a GTP bound “on” state and a GDP bound “off” state. It has been proposed that LRRK2 kinase activity may be increased upon GTP binding at the LRRK2 Ras of complex proteins (ROC) GTPase domain. Here we extensively test this hypothesis by measuring LRRK2 phosphorylation activity under influence of GDP, GTP or non-hydrolyzable GTP analogues GTPγS or GMPPCP. We show that autophosphorylation and lrrktide phosphorylation activity of recombinant LRRK2 protein is unaltered by guanine nucleotides, when co-incubated with LRRK2 during phosphorylation reactions. Also phosphorylation activity of LRRK2 is unchanged when the LRRK2 guanine nucleotide binding pocket is previously saturated with various nucleotides, in contrast to the greatly reduced activity measured for the guanine nucleotide binding site mutant T1348N. Interestingly, when nucleotides were incubated with cell lysates prior to purification of LRRK2, kinase activity was slightly enhanced by GTPγS or GMPPCP compared to GDP, pointing to an upstream guanine nucleotide binding protein that may activate LRRK2 in a GTP-dependent manner. Using metabolic labeling, we also found that cellular phosphorylation of LRRK2 was not significantly modulated by nucleotides, although labeling is significantly reduced by guanine nucleotide binding site mutants. We conclude that while kinase activity of LRRK2 requires an intact ROC-GTPase domain, it is independent of GDP or GTP binding to ROC.

## Introduction

Leucine rich repeat kinase 2 (LRRK2) has been identified as a Parkinson's disease (PD) gene responsible for parkinsonism with a clinical course essentially identical to that in idiopathic PD [1], [2]. LRRK2 encodes a 2527 amino-acid multidomain protein including several regions predicted to be involved in protein-protein interactions. Potential protein-protein interaction regions include an ankyrin repeat domain, a leucine rich repeat domain and a WD40 domain as well as two catalytic domains including a GTPase domain of the Ras of complex proteins family (ROC) and a kinase domain of the tyrosine kinase like family [3], [4]. Ras family GTPases and tyrosine kinase like kinases are often associated as elements of the same intracellular signaling pathway, suggesting a functional interaction between both of these catalytic functions within LRRK2. Ras GTPases act as molecular switches cycling between a guanosine triphosphate (GTP) bound ‘on’ state and a guanosine diphosphate (GDP) bound ‘off’ state. In the ‘on’ state, Ras GTPases activate an effector protein such as a kinase via direct binding.

For LRRK2, it has been suggested that LRRK2 kinase may be the downstream effector of LRRK2 ROC (reviewed in [5]). Indeed, functional mutant forms of LRRK2 in which guanine nucleotide binding is disrupted have been shown to display very low kinase activity suggesting that the ROC GTPase domain may regulate kinase activity [6], [7], [8]. Active or inactive states of Ras-GTPases can be mimicked *in vitro* using GDP for the inactive state and non-hydrolyzable GTP analogues such as guanosine - 5′ - O - [γ - thio] triphosphate (GTPγS) or guanosine - 5′ - [(β, γ) - methyleno] triphosphate (GMPPCP) for the active state. Enhanced LRRK2 autophosphorylation activity has been reported when the non-hydrolysable GTP analogue GTPγS was added to the kinase reaction [9], however the addition of GDP did not have an ‘off’ effect as would be expected. Although enhanced kinase activity has also been reported when GTPγS was added to the cellular lysate prior to protein purification [8], this finding could not be reproduced with recombinant protein in solution [10]. Therefore, although widely discussed, the data showing that GTP stimulates LRRK2 kinase activity is difficult to interpret as to whether this is direct binding and therefore a simple intramolecular switch mechanism or whether the mechanism is indirect.

Because varying results have been reported using different approaches, we sought to further elucidate the issue of how nucleotides bound to the ROC domain influence kinase activity. For this we compared several modes of application of guanine nucleotides to full length recombinant LRRK2 protein purified from HEK293T cells, coupled to measures of autophosphorylation as well as LRRK2-mediated phosphorylation of lrrktide, a specific *in vitro* peptide substrate [11]. Our data show that an intact ROC-GTPase domain is required for LRRK2 kinase activity and that kinase activity remains unchanged upon direct application of GDP compared to GTP or non-hydrolyzable GTP analogues, reconciling discrepancies in previous reports.

## Results

### LRRK2 kinase activity of the affinity purified protein is not altered upon co-incubation or preloading with guanine nucleotides

**Figure 1:**
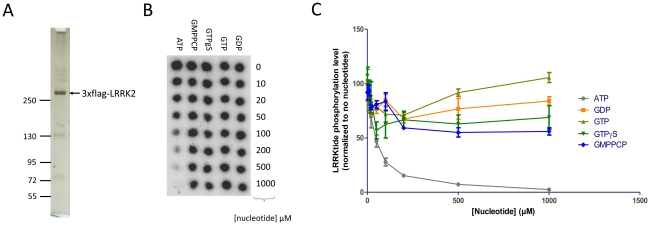
Kinase activity of recombinant LRRK2 protein when co-incubated with guanine nucleotides. A. Silver stain of SDS-PAGE gel of purified LRRK2 used in the kinase assays. B. Lrrktide phosphorylation by LRRK2 (30 minute incubation) was performed with co-incubation of varying concentrations of nucleotides as described in materials and methods. Shown is a representative autoradiogram of P81 filter spotted with P^32^ labeled lrrktide from the different assay conditions (*n* = 4). C. Quantification of lrrktide phosphorylation levels (y-axis) shown in panel B and plotted against the concentration of nucleotide co-incubated. Results in C were analzed by 2-way ANOVA as described in materials and methods, no significant differences were observed in the GTP, GTPγS or GMPPCP groups compared to the GDP group.

**Figure 2:**
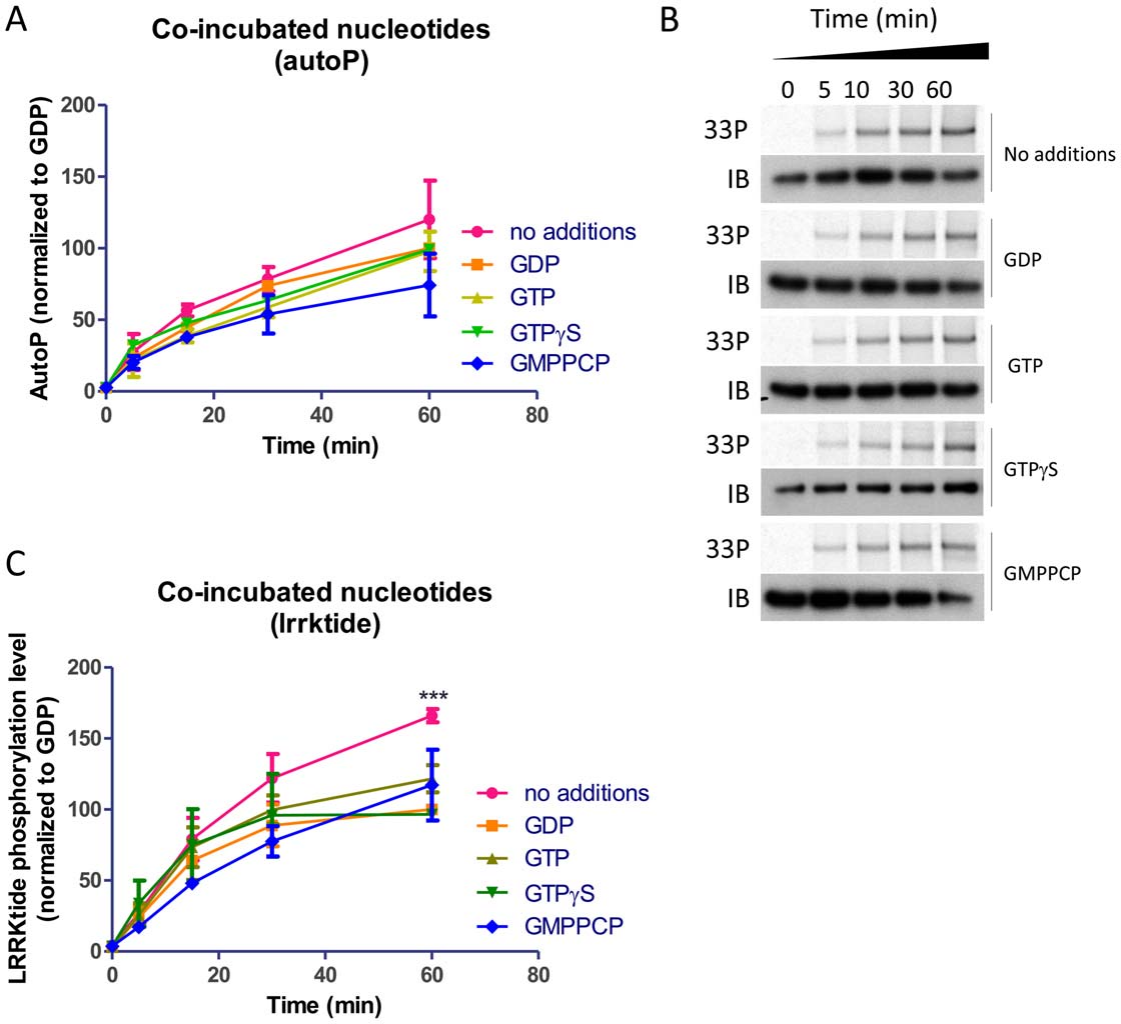
Time course of LRRK2 kinase activity when purified LRRK2 is co-incubated with guanine nucleotides. Purified LRRK2 was assayed for phosphorylation activity alone or in the presence of various guanine nucleotides. A. Time course of autophosphorylation of LRRK2 alone or co-incubated with 10 µM GDP, GTP, GTPγS or GMPPCP (*n* = 4). B. Representative autoradiograms and western blots of autophosphorylation samples from A. C. Time course of lrrktide phosphorylation activity of LRRK2 alone or co-incubated with 10 µM GDP, GTP, GTPγS or GMPPCP (*n* = 4). Results in A and C were analzed by 2-way ANOVA as described in materials and methods (*** P<0.001).

We first tested whether inclusion of nucleotides in the kinase reaction would alter LRRK2 kinase activity using purified soluble full-length LRRK2 protein (Figure 1A). Via metabolic labeling and thin layer chromatography analysis, we found that our stringent purification procedure yielded protein devoid of guanine nucleotides (supplementary figure S2). Co-incubation of LRRK2 with concentrations of guanine nucleotides varying from 0 to 1 mM did not alter LRRK2 mediated phosphorylation of the lrrktide peptide substrate (Figure 1B–C), while cold ATP was able to compete with radioactive ATP for lrrktide phosphorylation. The apparent Km_ATP_ was 41.73+/−1.42 µM, a value comparable to that obtained with truncated LRRK2 [12]. We also found that guanine nucleotides did not alter the time course of phosphorylation either for lrrktide phosphorylation or for autophosphorylation (Figure 2).

**Figure 3:**
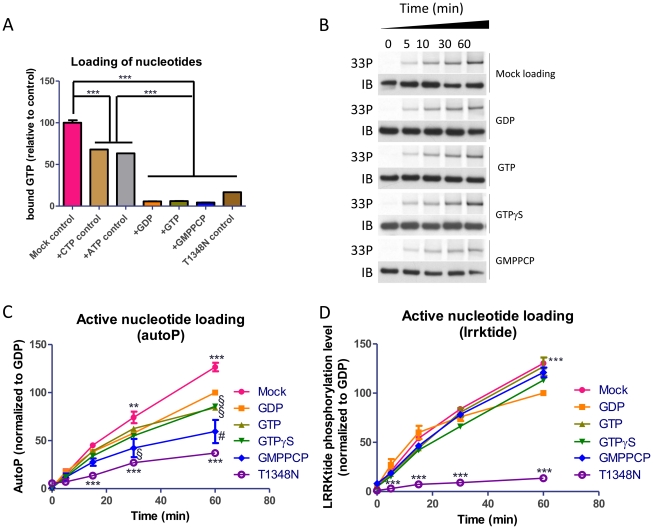
Effect of active preloading of guanine nucleotides to LRRK2 protein on kinase activity of LRRK2. A. Validation of the procedure to load LRRK2 with specific guanine nucleotides. Purified 3flag-LRRK2 bound to affinity resin was suspended in loading buffer and incubated at 30°C with GTP-α-P^32^ alone or with addition of 200 µM ‘cold’ nucleotides. After 1 h incubation, excess nucleotides were rinsed away and the amount of bound isotopic GTP was measured via scintillation counting and expressed as binding level relative to control protein (*n* = 3). Data were submitted to one-way ANOVA and post-hoc Dunnett test using control groups CTP-control, ATP-control and mock (*** P<0.001). B–C. Representative autoradiograms and western blots of autophosphorylation samples (B) and quantification (C) of the time course of autophosphorylation of LRRK2 following loading with different guanine nucleotides (*n* = 4). D. Time course of lrrktide phosphorylation activity of LRRK2 following loading with different guanine nucleotides (*n* = 4). Included in panels C–D are quantifications of the LRRK2 T1348N mutant autophosphorylation and lrrktide phosphorylation activities relative to LRRK2 wt showing reduced kinase activity for this GTP binding deficient mutant. Results in C and D were analzed by 2-way ANOVA as described in materials and methods (*** P<0.001). Symbols # (P<0.001) and § (P<0.01) denote significant differences for GTP, GTPγS or GMPPCP treatment groups which show reduced activity compared to the GDP control group.

In order to better control the nucleotide bound state of LRRK2, we prepared recombinant LRRK2 specifically preloaded with nucleotides via an *in vitro* loading procedure. In this procedure, purified proteins bound to the affinity resin are equilibrated in buffer containing an excess of nucleotides and incubated at 30°C to allow the loading of nucleotides to the GTP binding site. Unbound nucleotides are then washed away to yield a protein loaded with a specific nucleotide. The efficiency and specificity of the loading was tested using radioactively labeled GTP, which was completely outcompeted by an excess (200 µM) of various cold guanine nucleotides, while 200 µM ATP or CTP did not efficiently compete for GTP binding (Figure 3A). In addition, low binding levels were observed for the T1348N GTP-binding deficient mutant.

Recombinant protein prepared via this procedure and then eluted retained kinase activity both in autophosphorylation and in lrrktide phosphorylation. In these conditions, autophosphorylation was not significantly enhanced by GTP or GTP analogues compared to GDP. On the contrary, GTP or GTP analogues led to unchanged autophosphorylation levels or reduced autophosphorylation levels at the longer time points compared to GDP (Figure 3C). Lrrktide phosphorylation levels were not altered by GTP, GTPγS or GMPPCP compared to GDP (Figure 3D). At the longer time points GDP treated protein had a lowered kinase activity compared to mock treated protein (Figures 3C–D). By comparison, the LRRK2 GTP binding deficient mutant T1348N displayed very weak phosphorylation of lrrktide compared to wild type (Figure 3D) consistent with findings using autophosphorylation as a readout (Figure 3C and ref. [7]).

### LRRK2 kinase activity is modestly enhanced by application of GTPγS or GMPPCP to cell lysates prior to protein purification

**Figure 4:**
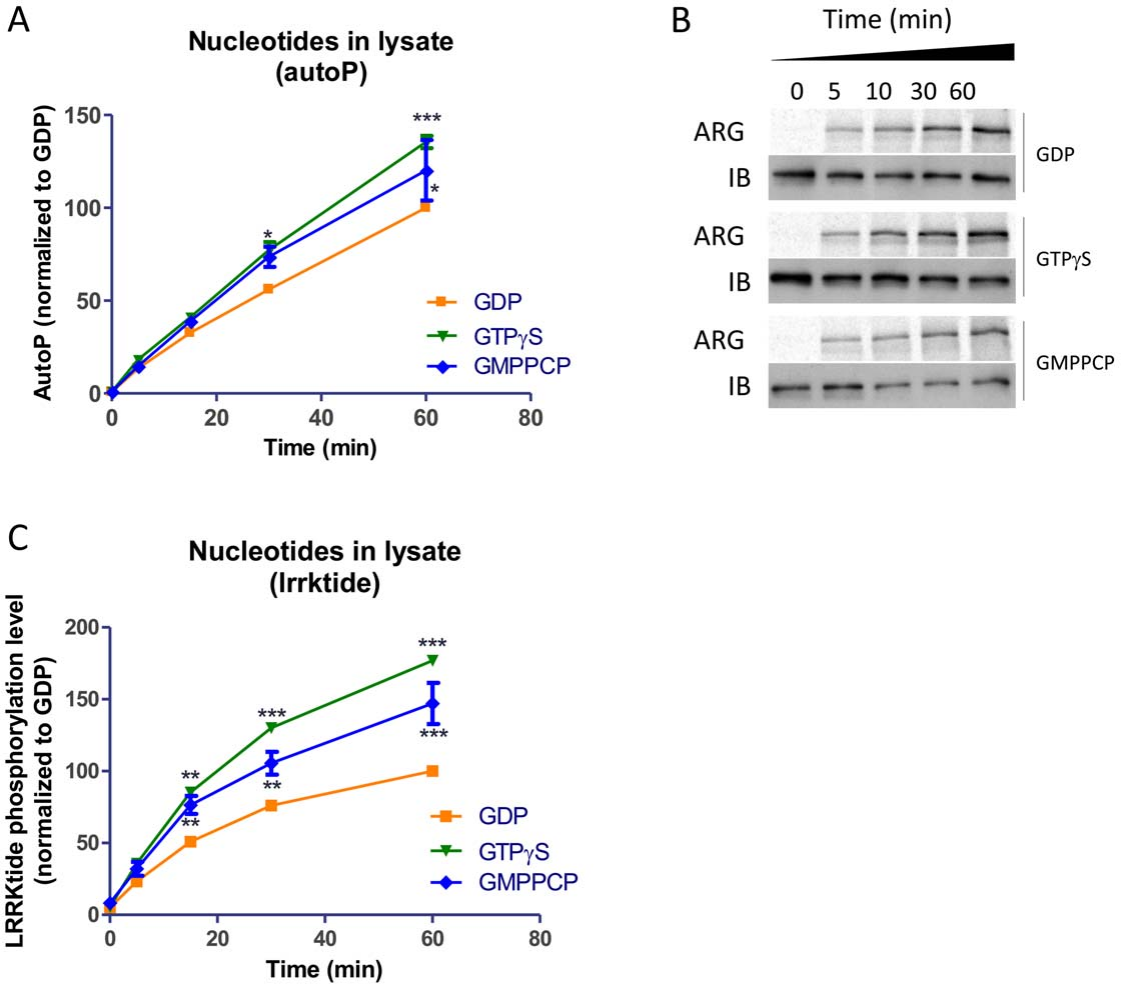
Kinase activity of LRRK2 purified from cell lysates treated with different guanine nucleotides. Cleared cell lysates were incubated with 10 µM of guanine nucleotides for 30 minutes at 30°C. Following this treatment, proteins were purified from treated lysates (all steps at 4°C) and kinase activity was monitored. A. Time course quantification of the autophosphorylation activity of LRRK2 protein prepared from treated cell lysates (*n* = 4). B. Representative autoradiograms and western blots of autophosphorylation samples. C. Time course quantification of LRRKtide phosphorylation levels of LRRK2 protein prepared from treated cell lysates (*n* = 6). Statistical differences of the GDP treatment compared to the GTPγS or GMPPCP treatment were tested by 2-way ANOVA. Differences revealed by the post test are indicated: * P<0.05, ** P<0.01, *** P<0.001.

In a third series of experiments, nucleotides GDP, GTPγS or GMPPCP were added to cell lysates expressing 3flag-LRRK2 and the lysate-nucleotide mix was incubated at 30°C for 30 minutes. Subsequently purified protein was tested for kinase activity by autophosphorylation assay (Fig. 4A–B) or lrrktide phosphorylation assay (Fig. 4C). 2-way anova analysis revealed a significant enhancement of activity in the GMPPCP and GTPγS groups compared to the GDP group. At 30 minutes incubation, the percent enhancement for lrrktide phosphorylation was 71.8+/−10.2 for GTPγS and 38.6+/−10.4 for GMPPCP while for autophosphorylation these values were +38.0+/−7,6% for GTPγS and +31.4+/−9.6 for GMPPCP (all values are mean +/− SEM).

### The macromolecular properties of purified LRRK2 are unchanged by guanine nucleotides

**Figure 5:**
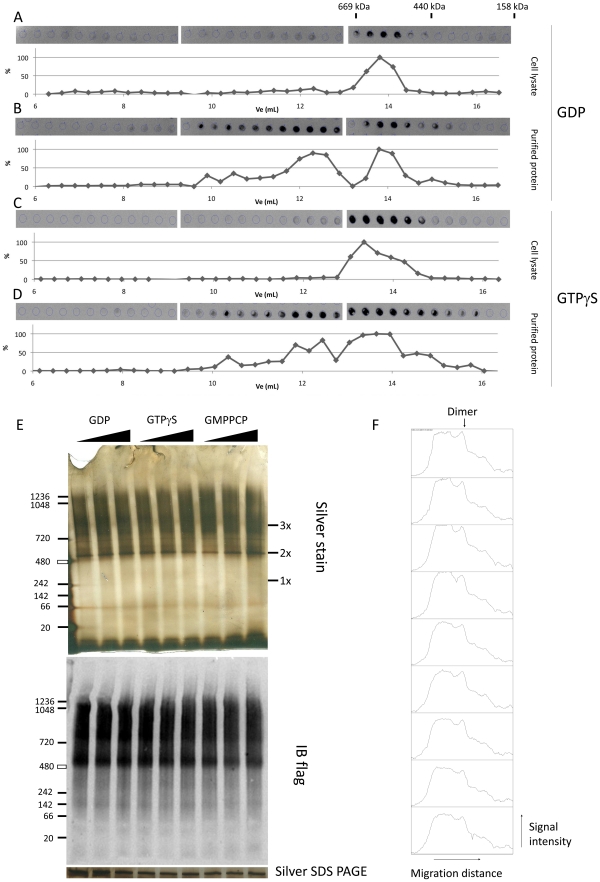
Influence of guanine nucleotides on macromolecular properties of LRRK2. A–D. Size exclusion chromatography elution profiles of LRRK2 in cell lysates or purified LRRK2 in the presence of GDP or GTPγS. 3xflag LRRK2 was expressed by transient transfection in HEK293T cells. Cleared cell lysates (A & C) or purified protein (B & D) were prepared as described in Materials and Methods and submitted to SEC in the presence of 10 µM of either GDP (A & B) or GTPγS (C & D). LRRK2 elution profiles were obtained by measuring LRRK2 levels in each elution fraction via immuno dot blot and are displayed as relative signal intensity in function of the elution volume, as described in Materials and Methods. The elution peaks of the protein standards are indicated above the dot blot of panel A. E–F. Native PAGE of LRRK2 purified from lysates loaded with different concentrations of nucleotides. 3xflag tagged LRRK2 was expressed in HEK293T cells via transient transfection. Cells were lysed at 48 h post-transfection and incubated with varying concentrations of GDP, GTPγS or GMPPCP (10, 100 and 500 µM) for 30 minutes at 30°C. Treated lysates were purified as described in materials and methods and separated via native PAGE. Gels were silver stained (top panel) or blotted onto PVDF membranes to detect flag immunoreactivity E. LRRK2 protein amounts visualized via silver staining on SDS-PAGE are shown under the native PAGE images. F. Signal intensity plotted against migration distance for each lane. The arrow marks the peak corresponding to the band which migrates at the predicted size of a LRRK2 dimer. Data are representative of 3 experiments.

Because the dimerization state of LRRK2 has been linked to its activity [6], [13], [14], we decided to test whether LRRK2 macromolecular properties were altered in the presence of GDP compared to GTPγS. For this, analytical size exclusion chromatography (SEC) was performed on cleared cell lysates of LRRK2 expressing cells or on purified LRRK2 using a column equilibrated with SEC buffer containing 10 µM of either GDP or GTPγS. The resulting chromatograms are given in Figure 5. These show that LRRK2 displays a peak corresponding to the apparent size of a dimer. Higher molecular weight peaks are observed in the purified protein samples while these higher molecular weight species are absent or very low abundant in the cleared cell lysates. However, GTPγS addition did not change the SEC profiles compared to GDP.

To confirm this with a second technique, we used Native PAGE analysis of purified LRRK2 protein following lysate treatment with either GDP, GTPγS or GMPPCP. All treatments displayed equivalent band profiles and were analogous to SEC results. In silver stained native PAGE gels as well as flag-M2 immunoblots of native gels, a band is observed around the expected size of the dimer with a smear of proteins at higher molecular weights until above 1.2 MDa (Figure 5E–F). Therefore, addition of non-hydrolyzable guanine nucleotides did not change the apparent molecular state of LRRK2.

### Nucleotide treatment does not alter 14-3-3 binding nor phosphorylation state of LRRK2

The enhanced LRRK2 kinase activity observed when GTP analogues are applied to the cell lysates points to the presence of LRRK2 cellular cofactors which can modulate LRRK2 activity under influence of guanine nucleotides. One recently reported interactor of LRRK2, 14-3-3 [15], [16] can be found in Ras-GTPase pathways [17]. Therefore, we tested whether guanine nucleotide treatment of LRRK2 would modulate the 14-3-3 binding. For this, we treated lysates expressing LRRK2 as described above and further purified LRRK2 under CoIP conditions. However we found that this treatment did not lead to altered 14-3-3 binding as measured by the CoIP assay (Supplementary figure S3). Next we assayed autophosphorylation levels of LRRK2 co-incubated with guanine nucleotides after co-immunoprecipitation, but no significant differences were observed (data not shown).

### Phosphorylation of LRRK2 in cells depends on an intact ROC GTPase domain

**Figure 6:**
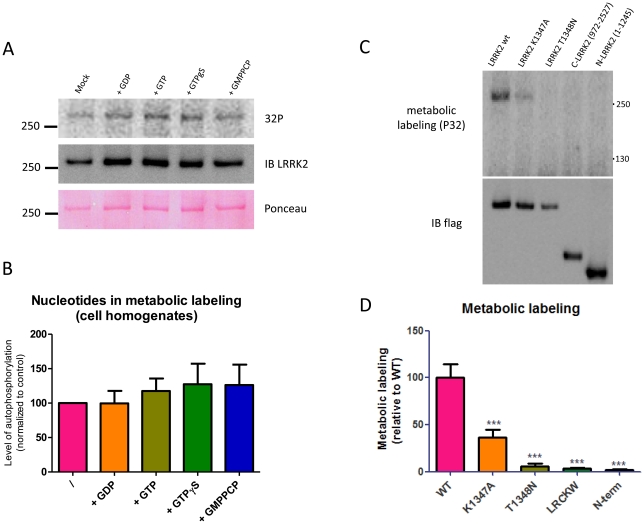
Metabolic labeling of LRRK2 depends on an intact ROC domain. A–B. Influence of guanine nucleotides on phosphorylation of LRRK2 in cellular lysates. HEK293T cells expressing LRRK2 were lysed and incubated with ATP-^32^P for 30 minutes at 30°C without additions (control) or in the presence of 10 µM GDP, GTP or the non hydrolyzable GTP analogues GTPγS and GMPPCP. LRRK2 was subsequently IP purified and submitted to SDS-PAGE and blotting to a PVDF membrane A. Shown here are the representative blot autoradiograms, immunoblot detection and ponceau staining of the phospholabeled samples. B. Quantification of A. C–D. Metabolic labeling of LRRK2 wt, GTP binding deficient LRRK2 mutants (K1347A, T1348N), and LRRK2 C-terminal (972–2527, encompassing ROC domain and lacking most cellular phosphorylation sites) and N-terminal (1–1245, encompassing cellular phosphorylation sites and lacking ROC domain) fragments. C. Representative blot autoradiograms and blot immunodetection of the metabolically labeled samples. D. Quantification of C. Data are representative of 4 experiments. Statistical differences of results in panels B and D were tested by one-way ANOVA as described in the materials and methods section. *** P<0.001.

It has been previously established that LRRK2 is phosphorylated in cells, with most sites clustering between residues 855 and 973 [7], [8], [18]. In order to examine whether nucleotides may influence the phosphorylation status of LRRK2 in cells, we performed metabolic labeling in intact cells and cell lysates. Figure 6A–B shows the incorporation of P^32^ in LRRK2 in normal cell lysates or after addition of GDP, GTP, GTPγS or GMPPCP. Although mean values were ∼20% higher for GTP and its analogues compared to GDP, these differences were not statistically significant.

We also performed metabolic labeling of LRRK2 proteins, showing that wild type LRRK2 efficiently incorporates phosphates in cells; however, the K1347A and T1348N GTP binding deficient mutations of the ROC domain did not (Fig. 6C–D). Labeling of the N-terminal (1–1245, construct encompassing the constitutive phosphorylation region [18] but lacking ROC and other C-terminal domains) and C-terminal (972–2527, construct encompassing LRR and other C-terminal domains, but lacking most constitutive phosphorylation sites) half sequences of LRRK2 was also undetectable.

## Discussion

The LRRK2 protein resembles a group of signaling cascade proteins tethered together in a single polypeptide chain. Indeed, Ras-GTPases homologous to LRRK2 ROC domain and tyrosine kinase like kinases homologous to the LRRK2 kinase domain are often found within the same signaling cascade. Signaling cascades of the Ras-GTPase family include both upstream and downstream kinases. For instance, in the case of Ras, a signaling cascade is initiated by ligand binding to a tyrosine kinase receptor which induces dimerization and autophosphorylation of the receptor [19]. This in turn leads to a series of events including the activation of src tyrosine kinase and the recruitment of accessory proteins that activate Ras protein by promoting GTP binding on Ras leading to a GTP bound form of Ras. Ras-GTP can bind its downstream effector Raf, a mitogen activated protein kinase kinase kinase, which is the first of a three tiered cascade of kinases. Therefore two hypotheses can be formulated to explain the potential interplay between ROC and kinase [5].

A first hypothesis is that the LRRK2 kinase domain is a downstream effector of ROC. In this scenario, kinase activity of LRRK2 is predicted to be turned on when ROC binds GTP and turned off when ROC binds GDP. We first tested this hypothesis by performing kinase assays on purified LRRK2 in the presence of varying concentrations of guanine nucleotides. However, the addition of GTP or non-hydrolyzable GTP analogues GTPγS or GMPPCP to purified LRRK2 did not significantly enhance LRRK2 activity in the lrrktide assay. Conversely, addition of GDP to the kinase reaction mix had no inhibitory effect on LRRK2 phosphorylation activity, indicating that GDP does not provide an off switch to LRRK2 kinase activity in these conditions. Also, the fact that guanine nucleotides are washed off of the protein in our purification protocol shows that guanine nucleotides are not a requirement for kinase activity. These observations are consistent with results obtained with a similar full length protein purified from a mouse expression system [10].

One potential caveat of testing effects of guanine nucleotides on kinase activity by simple addition of nucleotides to the enzymatic reaction is that two processes are occurring at the same time, namely *in vitro* phosphorylation and guanine nucleotide exchange. In order to dissociate these two processes, we performed an *in vitro* guanine nucleotide exchange such that proteins tested are a homogeneous mix of protein saturated with a specific guanine nucleotide at the moment of kinase activity testing. This loading step is commonly applied to Ras family GTPases to test ‘on’ and ‘off’ states, for example for the Ras family GTPases Rac or cdc42 whose binding to p21 activated kinase 1 (pAK1) occurs exclusively in the GTP-bound state [20]. In these conditions where LRRK2 is saturated by specific nucleotides, we also found that GTP or analogues did not enhance and GDP did not inhibit LRRK2 kinase activity. It should be noted that loading of GTP or non-hydrolyzable GTP analogues slightly but significantly reduced LRRK2 autophosphorylation activity compared to GDP loading, while this is not the case for the lrrktide readout. This discrepancy may be explained by the fact that autophosphorylation occurs at sites influenced by GTP binding while lrrktide is not influenced by guanine nucleotides. Lrrktide is derived from moesin, which does not bind guanine nucleotides [11]. The main autophosphorylation sites of LRRK2 are found in the guanine nucleotide binding domain of LRRK2 (ROC) [21]. Since Ras family proteins adopt different confirmations when different nucleotides are bound [22], such as in the active loading procedure performed in this experiment, these different conformations may influence the phosphorylation in this region, although structural biology experiments would have to be performed to explore this.

The lack of enhancement of LRRK2 kinase activity by loading of GTP is in contrast with the greatly reduced kinase activity measured for GTP binding deficient mutants K1347A or T1348N [7], [8]. Recently T1348 has been reported to be an autophosphorylation site of LRRK2 [18], [21] and may account for some reduction in autophosphorylation activity. However, the observed reduction in lrrktide phosphorylation activity for the T1348N mutant clearly allow us to conclude that the T1348N inhibits kinase activity. Taken together, these data show that the capacity for GDP/GTP binding is essential for LRRK2 kinase activity, but that the specific guanine nucleotide bound to LRRK2 ROC does not play a modulatory role on LRRK2 kinase activity. Therefore LRRK2 kinase domain is not the direct effector of LRRK2 ROC domain.

Previous reports using GTP-beads as affinity resin have shown that GDP or GTP binding occurs very efficiently in lysates [8], [23]. Therefore, we also tested whether application of guanine nucleotides to LRRK2 expressing cell lysates could modulate LRRK2 kinase activity. Interestingly, we found that LRRK2 purified after lysate treatment with GTPγS or GMPPCP showed a modest but significant enhancement of autophosphorylation and lrrktide phosphorylation compared to lysate treatment with GDP. Since nucleotides have no differential effect on kinase activity when applied to purified protein, these data point to an activation of LRRK2 through another guanine nucleotide dependent mechanism, for instance via an upstream guanine nucleotide binding protein which may activate LRRK2 in a GTP-dependent manner.

Our results are thus consistent with the conclusion that LRRK2 kinase is not the effector of ROC. This observation would also be consistent with the alternative hypothesis that kinase is a modulator of ROC function. Supporting this possibility, LRRK2 kinase phosphorylates its own ROC domain [18], [21]. If LRRK2 kinase is not the effector of ROC, this implies that the ROC downstream effector is still unknown. Following the logic of the on/off switch, it will be necessary to search for cellular binding partners of ROC in its GTP bound state in order to identify this (or these) potential effector(s). The identification of GTP dependent binding partners of LRRK2 will provide a valuable step forward in the elucidation of the LRRK2 signaling pathway.

One possibility is that guanine nucleotides regulate the binding of LRRK2 with itself. Indeed, LRRK2 can self interact and analysis of native protein preparations shows that LRRK2 resides primarily in dimer sized complexes [6], [13], [14], [24], [25]. Also, results from a prokaryotic ROCO family homolog of LRRK2 suggest a possible regulation of dimerization by guanine nucleotides [26], [27], although this has not yet been assessed in LRRK2. Data presented here (Figure 6) using SEC and native PAGE analysis show that guanine nucleotides do not differentially affect macromolecular properties of LRRK2. One striking observation is that while both purified LRRK2 and LRRK2 in cell lysates show dimer sized peaks, the purified protein displays relatively more high molecular weight bands compared to LRRK2 in cell lysates. This indicates that LRRK2 oligomeric complexes are regulated by cellular binding partners such as chaperones.

Several proteins have previously been reported to bind to LRRK2 in eukaryotic cells including cytoskeletal protein such as tubulins [28], [29] or F-actin [30], HSP70 [31] and 14-3-3 proteins [15], [16]. The binding between LRRK2 and tubulins has been shown to be guanine nucleotide independent, although the effect of guanine nucleotides has not been tested for most other LRRK2 interactors reported to date. 14-3-3 proteins have also been implicated in processes regulated by guanine nucleotides, for instance 14-3-3 is phosphorylated by the Ras-GTPase effector pAK1 [17] and binds to the guanine nucleotide exchange factor Tiam1 [32]. Our results show that LRRK2 binding to 14-3-3 is not altered by GDP or GTPγS (supplemental figure S3), indicating that 14-3-3 is probably not found upstream in the GTP dependent activation of LRRK2.

Finally, we also tested for a link between guanine nucleotides and cellular phosphostatus of LRRK2. LRRK2 can be phosphorylated at multiple sites [18], [21], [33], with ‘constitutive’ phosphorylation sites found in the region preceding the leucine rich repeat domain (located between S850 and S979) [8], [16], [18]. Studies using kinase inhibitors directed against LRRK2 have shown that the cellular phosphorylation of LRRK2 in this region is regulated by its kinase activity via feedback regulation to a second kinase [15], [16], [34]. Our experiments confirm that LRRK2 is readily phosphorylated in cells, however we show that cellular phosphostatus of LRRK2 is not altered by treatment in cell lysates for one guanine nucleotide compared to another. As 14-3-3 binding has been shown to be regulated by the degree of the cellular phosphorylation of LRRK2 [15], [16], this lack of effect of guanine nucleotides on 14-3-3 binding correlates to the findings that guanine nucleotides do not alter the phosphorylation state of LRRK2 (figure 6B). As for the *in vitro* data, this finding contrasts with data from GTP binding deficient mutants (K1347A, T1348N) for which phospholabeling is quite inefficient (Figure 6). Interestingly, simple overexpression of the cellular phosphorylation sites but without C-terminal sequences including ROC (amino acids 1–1245), is not sufficient for these sites to be phosphorylated in the cell. Therefore, we can conclude that constitutive phosphorylation of LRRK2 in cells requires the presence of an intact ROC GTPase domain; however it is not significantly modulated by GDP or GTP.

In summary, the present study illustrates that the nature of the guanine nucleotide bound to LRRK2 has little influence on LRRK2 kinase activity, although the capacity for guanine nucleotide binding *per se* is crucial for this function. Similarly, an intact ROC domain is required for phospholabeling of LRRK2 in cells while the specific guanine nucleotide bound form of ROC does not alter the phosphostatus of LRRK2. These findings effectively reconcile results obtained using functional mutants of LRRK2 with results obtained from manipulation of the nucleotide bound state of LRRK2. These results further show that the downstream effector(s) of LRRK2 ROC has yet to be identified.

## Materials and Methods

### Reagents

The pCHMWS-3xflag-LRRK2 eukaryotic expression plasmids are described in reference [23]. The DR4A/3EDD anti-LRRK2-kinase domain antibody is described in reference [35] and goat polyclonal anti 14-3-3 antibody was purchased from Santa Cruz (Santa Cruz Biotechnology, Santa Cruz, CA, USA). Lrrktide peptide [11] was synthesized by Enzo life sciences.

### Expression and purification of recombinant LRRK2 protein

HEK293T cells (ATCC CRL-11268) were transfected with pCHMWS-3xflag-LRRK2 plasmid using polyethyleneimine and lysed after 48–72 hours in lysis buffer (Tris 20 mM pH 7.5, NaCl 150 mM, EDTA 1 mM, Triton 1%, Glycerol 10%, protease inhibitor cocktail (Roche, Vilvoorde, Belgium)). Lysates were cleared by centrifugation at 20.000 g for 10 minutes and incubated with normal mouse IgGs bound to agarose beads (Sigma, Bornem, Belgium) to remove proteins aspecifically binding to agarose or mouse IgGs. After removal of the IgG bead slurry, lysates were incubated for 3 to 18 hours with flagM2 bound to agarose beads (Sigma). Beads were washed 4 times with wash buffer (Tris 25 mM pH 7.5, NaCl 400 mM, Triton 1%) and rinsed in kinase buffer (Tris 25 mM pH 7.5, MgCl_2_ 10 mM, dithiothreitol (DTT) 2 mM, Triton 0,02%, beta-glycerophosphate 5 mM, Na_3_VO_4_ 0.1 mM). For those assays using protein in solution, proteins were eluted in 5 volumes of kinase buffer containing 100 µg/ml 3xflag peptide (Sigma). For assays using purified protein bound to affinity resin, affinity beads were resuspended in an equal volume of kinase buffer unless otherwise indicated. Purity and concentration were assessed by SDS-PAGE (3–8% tris-acetate SDS gel, Invitrogen, Merelbeke, Belgium) and coomassie brilliant blue staining (Thermo Scientific, Hampton, NH, USA) or silver staining as shown in figure 1A (LRRK2 T1348N displayed purity comparable to LRRK2 wt (data not shown), as we previously reported [23]).

### *In vitro* phosphorylation assays

To assay autophosphorylation, eluted purified proteins or a suspension of affinity resin bound protein were incubated with 6 µCi of ^33^P-ATP or ^32^P-ATP (3000 Ci/mmol; Perkin Elmer) and 10 µM ATP per 40 µl reaction for 5–60 minutes at 30°C. Guanine nucleotides are also added to some kinase reactions to final concentrations as indicated in the results section. Reactions were terminated by adding 6× SDS loading buffer A (for eluted protein, composition: Tris 150 mM pH 6.8, 0.1% SDS, 30% glycerol, 120 µg/ml bromophenol blue, 10% beta-mercaptoethanol) or 2× SDS loading buffer B (for proteins bound to affinity resin, composition: Tris-HCl 160 mM pH 6.8, SDS 2%, DTT 0.2 M, glycerol 40%, bromophenol blue 2 mg/ml). Samples were loaded onto pre-cast Tris-acetate 3–8% gels (Invitrogen) or Tris-glycine 4–20% gels (Bio-Rad, Hercules, CA, USA) and transferred onto polyvinylidene fluoride (PVDF) membranes. Incorporated ^33^P or ^32^P was detected by autoradiography using a Storm 840 phosphorescence scanner (GE Healthcare). The same membranes were stained with Ponceau S (Sigma) to correct for protein loading and probed with DR4A/3EDD in house anti-LRRK2 kinase domain antibody [35] to confirm the presence of LRRK2. Densitometric analysis of the bands on the blot autoradiograms and immunoreactivity were performed using Aida analyzer v1.0 (Raytest, Straubenhardt, Germany) or ImageJ software (NIH, USA). Autophosphorylation levels were calculated as the ratio of the autoradiographic signal over the immunoreactivity level.

In enzymatic reactions testing lrrktide phosphorylation, reactions were prepared as described for autophosphorylation above with the addition of 200 µM lrrktide. Reactions were incubated at 30°C and stopped after 5–60 minutes by the addition of 500 mM EDTA containing bromophenol blue. Guanine nucleotides are also added to some kinase reactions to final concentrations as indicated in the results section. All reactions are carried out in the presence of 10 µM ‘cold’ ATP with the exception of the experiment testing varying concentrations of ATP (concentrations as indicated in the results section). Reactions were spotted to P81 phosphocellulose paper (Whatmann) and washed 4 times 10 minutes in 75 mM phosphoric acid. Lrrktide phosphorylation levels were measured via scintillation counting or via autoradiography [36]. Kinase assays were performed for each condition using at least three independent protein preparations.

### Nucleotide loading

For experiments in which LRRK2 was loaded with specific guanine nucleotides, affinity resin bound protein was washed as above, rinsed in loading buffer (Tris 25 mM pH 7.5, NaCl 150 mM, EDTA 5 mM, Triton 0.02%) and incubated with an excess (200 µM) GDP or GTPγS for 30 minutes at 30°C under light shaking [20], [37]. Nucleotide exchange was stopped and excess nucleotides removed by rinsing beads 3 times in kinase buffer. Validation of the loading procedure was performed using the same protocol with radioactively labelled GTP (GTP-α-^33^P), in the presence or absence of 200 µM ‘cold’ nucleotides.

### Co-immunoprecipitation (Co-IP)

HEK293T cells were transfected with pCHMWS-3flag-LRRK2 with polyethyleneimine and lysed after 48–72 hours in Co-IP buffer (50 mM Tris/HCl pH 7.5, 150 mM NaCl, 1 mM Na_2_EDTA, 1 mM PMSF, 0.1% Triton X-100, 10% glycerol, protease inhibitor cocktail (Roche) and phosphatase inhibitor cocktail (Roche). Lysates were centrifuged at 4°C for 10 minutes at 20.000 g and supernatant further cleared by incubation with normal mouse IgGs bound to agarose beads at 4°C with end over end mixing. After removal of the IgG beads by centrifugation, cleared lysates were incubated for 3 to 18 hours with flag M2 beads at 4°C. Beads were washed 4 times with Co-IP buffer. After four washes, immunoprecipitates were eluted by addition of 2× SDS loading buffer B. Samples were resolved on 3–8% tris-acetate gels. For detection of the 14-3-3 interaction, gels were blotted onto PVDF membranes and probed with goat anti 14-3-3 antibody (Santa Cruz). For those Co-IP preprations further submitted to *in vitro* autophosphorylation assay, beads were rinsed in kinase buffer, resuspended in equal volumes of kinase buffer and submitted to autophosphorylation in the presence or absence of nucleotides as described under ‘kinase assays’.

### Metabolic labeling

For labeling in intact cells, LRRK2 or LRRK2 fragments were expressed via transient transfection in HEK293T cells. At 48–60 hours post-transfection, cells were rinsed two times in DMEM without phosphates then metabolically labeled with 5 µCi/cm^2^ orthophosphate-P^32^ (Perkin-Elmer) in DMEM without phosphates at 37°C. Following labeling, cells were lysed and LRRK2 was immunoprecipitated using flag-M2 agarose beads. Immunoprecipitated protein was resolved on 3–8% SDS-PAGE gels and blotted to PVDF membranes. Membranes were processed as described above for the autophosphorylation assay. To identify nucleotides to LRRK2, nucleotides were eluted from purified LRRK2 protein in kinase buffer with an excess of GTP (1 mM), then separated by thin-layer chromatography (TLC) (Merck, Darmstadt, Germany) and visualized by autoradiography using a Storm 840 phosphorescence scanner (GE Healthcare).

For metabolic labeling of LRRK2 in cell lysates, LRRK2 was expressed in HEK293T cells as described above and lysed in kinase buffer (see above) with EDTA-free protease inhibitor cocktail (Roche), phosphatase inhibitor cocktail (Sigma) and 0,1% triton. Lysates were cleared by centrifugation at 20.000 g for 10 minutes and by incubation with normal mouse IgGs bound to agarose beads (Sigma). The cleared lysate was pooled and distributed into 5 tubes (500 µl lysate per tube). 20 µCi ATP-P32 was added to each tube, either without further additions (control reaction) or with addition of 10 µM of GDP, GTP, GTPγS or GMPPCP. Reactions were incubated at 30°C for 30 minutes under light shaking to keep protein in suspension. After incubation, the pan-kinase inhibitor staurosporine was added to a final concentration of 100 nM to halt kinase phosphorylation processes and lysates were incubated with flag-M2 affinity resin for 1–2 hours at 4°C. After washing affinity resin bound protein 4 times in IP wash buffer, 2× SDS loading buffer B was added and analyzed using SDS-PAGE as described above for the autophosphorylation assay. The labeling was repeated using at least three independent protein preparations per condition tested.

### Analytical size-exclusion chromatography (SEC)

SEC was performed on cleared cell lysates as well as on purified protein. 3xflag-LRRK2 was expressed in HEK293T cells as described above. Cleared cell lysates were made by lysis of cells in SEC lysis buffer (25 mM Tris pH 7.4, 150 mM NaCl, 5 mM MgCl2, 0.1% Triton, 1 mM DTT, protease inhibitor cocktail (Roche)) then clearing via centrifugation at 20.000 g for 10 minutes followed by clearing with normal mouse IgGs bound to agarose beads. Lysates were supplemented with either GDP or GTPγS 10 µM before loading onto the column. Purified protein for SEC analysis was obtained as described above using flag-M2 agarose beads, with the exception that proteins were eluted in SEC running buffer (25 mM Tris pH 7.4, 150 mM NaCl, 5 mM MgCl2, 0.02% Triton, 1 mM DTT) containing 100 µg/ml 3xflag peptide supplemented with 10 µM of either GDP or GTPγS.

Analysis was performed using a Superose 6 10/300 GL column (GE Healthcare) coupled to an AKTA purifier 10 UPC-900 system (GE Healthcare). The column was calibrated using protein standards (Gel Filtration Calibration Kit HMW, GE Healthcare: thyroglobulin (669 kDa), ferritin (440 kDa), aldolase (158 kDa) and ovalbumin (44 kDa)) in 50 mM Tris pH 7.5, 100 mM KCl, 5% glycerol (supplemental figure S1). Before analysis, the column was equilibrated in SEC running buffer containing 10 µM of either GDP or GTPγS. SEC runs were performed at 4°C with 100 µl cell lysate or purified protein sample. Fractions (300 µl) were analyzed via dot blotting (Bio-Dot Microfiltration Apparatus, Bio-Rad) onto nitrocellulose membrane (Bio-Rad) and detecting fractions immunoreactive to flag-M2 antibody. Signals were quantified by densitometry using Aida analyzer v1.0 (Raytest, Straubenhardt, Germany) and the elution profile was plotted as a percentage of the maximum signal. Molecular weights and Stokes radii were calculated from the standard curve obtained from the elution volumes of the standard proteins (supplementary figure S1), showing a resolution sufficient to discern alterations of 50–100 kDa in size.

### Immunoblotting procedures

Protein samples were resolved by electrophoresis on 3–8% tris-acetate (Invitrogen) or 4–20% tris-glycine (Bio-Rad) gradient gels, and electroblotted to PVDF membranes. Membranes were blocked with 5% skimmed milk (w/v) in 50 mM Tris/HCl, pH 7.5, 0.15 M NaCl and 0.1% (v/v) Tween 20 (TBST Buffer). Antibodies were used at 1∶10.000 in 5% (w/v) milk in TBST. Detection of immune-complexes was performed using horseradish-peroxidase-conjugated secondary antibodies (Dako, Heverlee, Belgium) and an enhanced-chemiluminescence reagent (Bio-Rad).

### Statistics

For the statistical comparisons, test values were normalized to control (for instance the GDP group). In the dose range experiment (Figure 1C), changes in kinase activity in the guanine nucleotide groups was tested for by 2-way ANOVA with concentration and treatment as factors followed by a Bonferroni post test for each concentration using GDP as the control group. In the time course experiments (Figures 2, 3, 4), changes in kinase activity in the test groups compared to the GDP control group was tested for by 2-way ANOVA with time and treatment as factors followed by a Bonferroni post test for each time point. In other experiments, values from test groups were tested for significant differences from the control group using a one-way ANOVA followed by a Dunnett post-hoc test. Statistical significance was set at p<0,05.

## Supporting Information

Figure S1 Calibration of size exclusion column. A. Chromatographic calibration curve for the standard proteins on Superose 6 10/300 GL column. The retention volume (V_e_) of thyroglobulin (669 kDa), ferritin (440 kDa), aldolase (158 kDa) and ovalbumin (44 kDa) was determined from the A_280 nm_ elution profile. Blue dextran was used to determine the void volume (V_0_) of the column (*not shown*). B. The experimental and calculated parameters for the equilibration of the Superose 6 10/300 GL column, with the apparent molecular weight (M_W_), the elution volume (V_e_), the void volume (V_o_), the gel phase distribution coefficient (K_av_ = (V_e_−V_0_)/(V_t_−V_0_), where V_t_ is the total column bed volume) and the Stoke's radius. C. Calibration curve displaying the relationship between Ln(M_w_) and V_e_/V_o_ obtained with the standard proteins as run on Superose 6 10/300 GL column. (D.) Calibration curve displaying the relationship between the Stokes radius and the √(−log(K_av_)) obtained with the standard proteins as run on Superose 6 10/300 GL column.
(TIF)

Figure S2 Analysis of guanine nucleotide bound to LRRK2 as purified in this study. LRRK2 constructs (as in figure 6) were metabolically labeled with [32P]-orthophosphate and submitted to the affinity purification procedure described in the materials and methods. Thin-layer chromatographic analysis of bound nucleotides for LRRK2 wt, LRRK2 K1347A, LRRK2 T1348N and N-terminal and C-terminal fragments shows that the purification procedure washes out all nucleotides.
(TIF)

Figure S3 Evaluation of the effect of guanine nucleotides on the binding of 14-3-3 to LRRK2. Displayed is the western blot detection of 14-3-3 protein co-immunoprecipitated with 3flag-LRRK2 following treatment of cell lysates with different guanine nucleotides. Representative of 2 experiments.
(TIF)
