# Supplementary figures and images for: LRRK2 Kinase Activity Is Dependent on LRRK2 GTP Binding Capacity but Independent of LRRK2 GTP Binding
Source: PLoS One. 2011 Aug 12;6(8):e23207. doi: 10.1371/journal.pone.0023207 (PMC3155532; doi:10.1371/journal.pone.0023207)

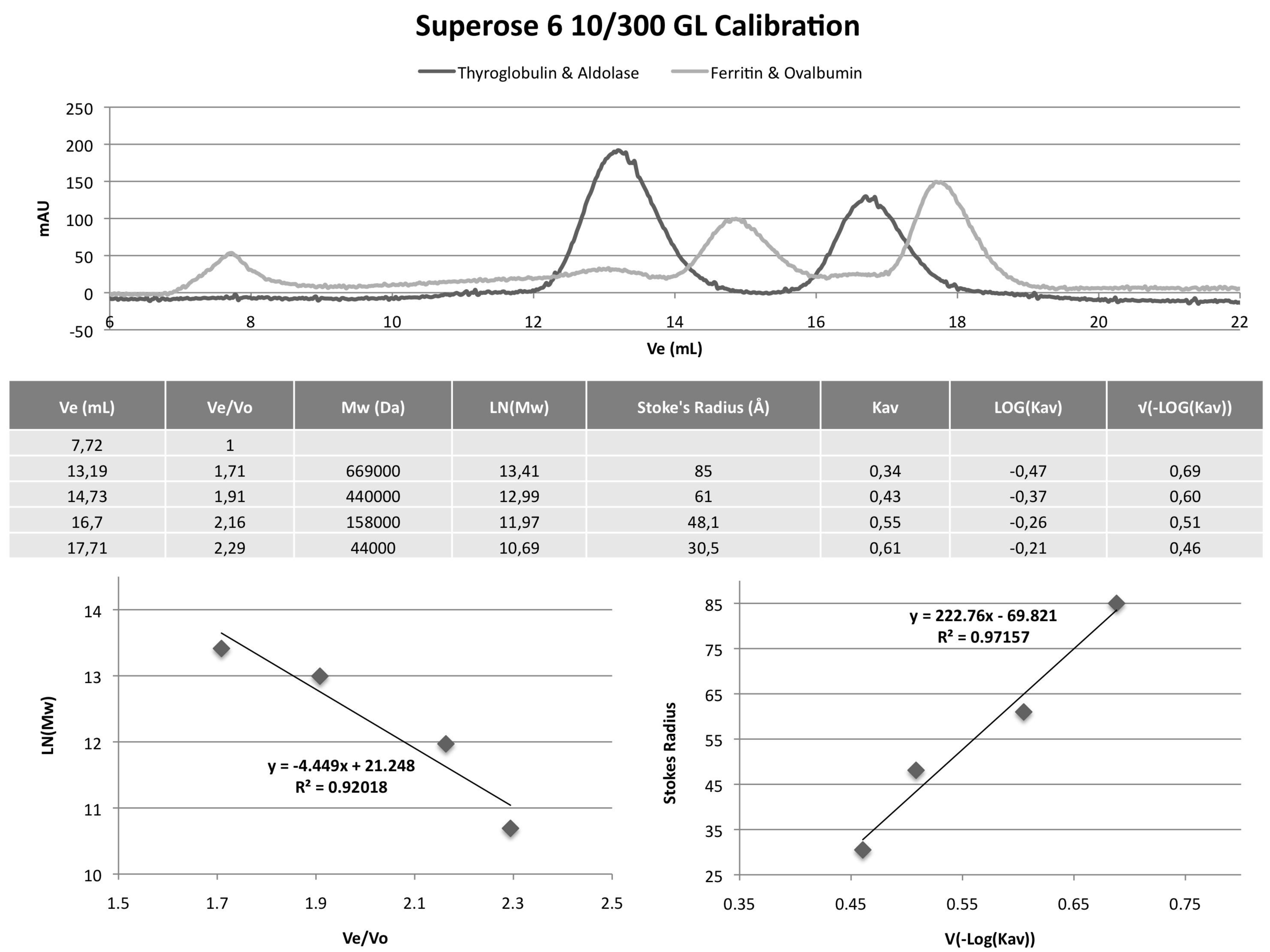

Supplement: Figure S1 — Calibration of size exclusion column. A. Chromatographic calibration curve for the standard proteins on Superose 6 10/300 GL column. The retention volume (Ve) of thyroglobulin (669 kDa), ferritin (440 kDa), aldolase (158 kDa) and ovalbumin (44 kDa) was determined from the A280 nm elution profile. Blue dextran was used to determine the void volume (V0) of the column (not shown). B. The experimental and calculated parameters for the equilibration of the Superose 6 10/300 GL column, with the apparent molecular weight (MW), the elution volume (Ve), the void volume (Vo), the gel phase distribution coefficient (Kav = (Ve−V0)/(Vt−V0), where Vt is the total column bed volume) and the Stoke's radius. C. Calibration curve displaying the relationship between Ln(Mw) and Ve/Vo obtained with the standard proteins as run on Superose 6 10/300 GL column. (D.) Calibration curve displaying the relationship between the Stokes radius and the √(−log(Kav)) obtained with the standard proteins as run on Superose 6 10/300 GL column. (TIF) [file pone.0023207.s001.tif]

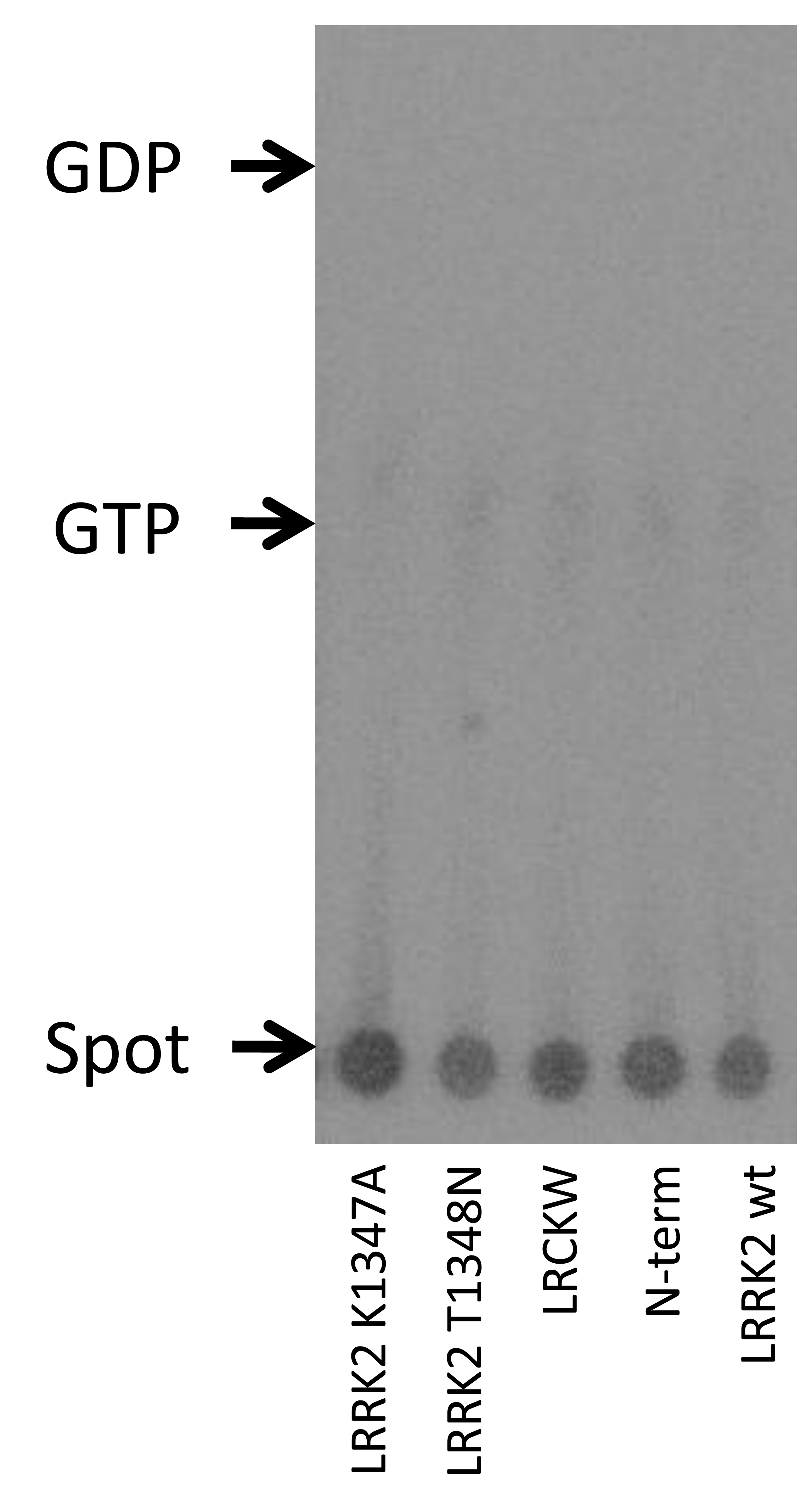

Supplement: Figure S2 — Analysis of guanine nucleotide bound to LRRK2 as purified in this study. LRRK2 constructs (as in figure 6) were metabolically labeled with [32P]-orthophosphate and submitted to the affinity purification procedure described in the materials and methods. Thin-layer chromatographic analysis of bound nucleotides for LRRK2 wt, LRRK2 K1347A, LRRK2 T1348N and N-terminal and C-terminal fragments shows that the purification procedure washes out all nucleotides. (TIF) [file pone.0023207.s002.tif]

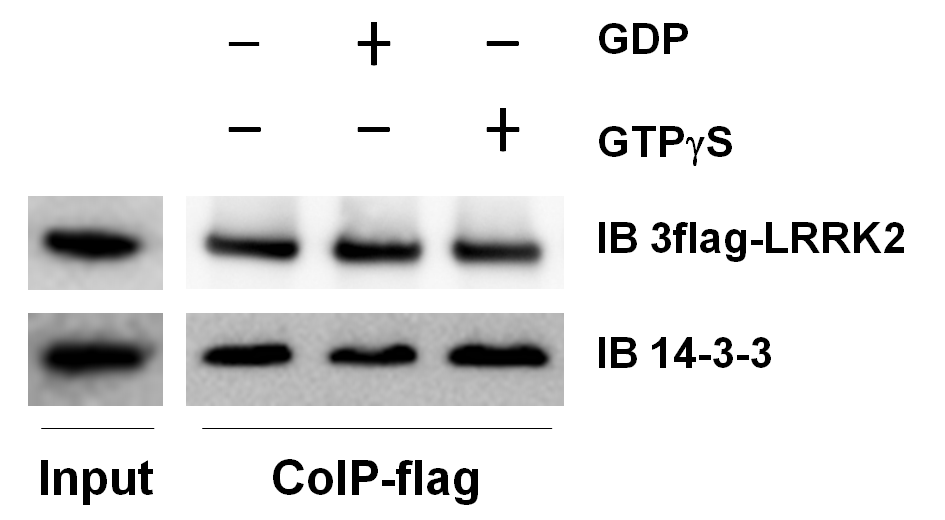

Supplement: Figure S3 — Evaluation of the effect of guanine nucleotides on the binding of 14-3-3 to LRRK2. Displayed is the western blot detection of 14-3-3 protein co-immunoprecipitated with 3flag-LRRK2 following treatment of cell lysates with different guanine nucleotides. Representative of 2 experiments. (TIF) [file pone.0023207.s003.tif]
